# Cognitive Skills of Young Children with and without Autism Spectrum Disorder Using the BSID-III

**DOI:** 10.1155/2011/759289

**Published:** 2011-02-23

**Authors:** Carolyn Long, Matthew J. Gurka, James Blackman

**Affiliations:** ^1^Hospital Education Program, University of Virginia Health System, Charlottesville, VA 22908, USA; ^2^Kluge Children's Rehabilitation Center and Research Institute, University of Virginia Children's Hospital, 2270 Ivy Road, Charlottesville, VA 22903, USA; ^3^Department of Community Medicine, West Virginia University, WV 26506, USA; ^4^Department of Pediatrics, University of Virginia, VA 22908, USA

## Abstract

*Objective*. The purpose of the study was to compare the cognitive skills of young children diagnosed with autism spectrum disorder (ASD) to same-aged peers referred for possible developmental delays or behavioral concerns using the *Bayley Scales of Infant Development-Third Edition*. *Method*. A retrospective chart review was conducted of 147 children ages 16 to 38 months who were referred to a diagnostic clinic for developmental evaluation. Children with ASD were compared to those without ASD with respect to cognition and language outcomes, both overall and by age. *Results*. While language skills in children with ASD were more significantly delayed than language skills in children without ASD, there was less discrepancy in the cognitive skills of children with and without ASD. *Conclusion*. Formal cognitive assessment of children with ASD can provide guidance for developmental expectations and educational programming. Cognitive skills of children with ASD may be underappreciated.

## 1. Introduction

The number of children diagnosed with autism spectrum disorder (ASD) has rapidly increased in recent years. Best estimate of current prevalence of children with ASD is just over one per 100 [1], and males are four times more likely to be diagnosed than females. Early diagnosis is recommended for effective intervention services for children and families. Comprehensive developmental assessment may assist in differential diagnosis and educational programming. While the presence of language delay has always been an essential component in the diagnosis of children with ASD, there has been less agreement on the cognitive ability of these children. 

Autism was first described by Kanner [2], who observed a number of children with characteristics that included obsessiveness, stereotypy, and echolalia, but exhibited “good cognitive potentialities.” Even in the children who had not developed language, Kanner noted an ability to perform tasks such as puzzles at or above age level. He reported, however, that “Binet or similar testing could not be carried out because of limited accessibility” [3].

The prevalence of intellectual disability (mental retardation or global developmental delay) in children with autism was estimated to be 90% before 1990 [4]. Prevalence studies since the year 2000 report rates of comorbidity of intellectual disability and autism at approximately 50% [5]. While intellectual disability has never been a component of the diagnostic criteria for autism, an associated diagnosis of intellectual disability ranging from mild to profound was noted by the authors of the *Diagnostic and Statistical Manual of Mental Disorder-IV* with 70% to 75% of children having both [6]. Edelson [7] conducted a systematic review of articles published between 1937 and 2003 that reported the prevalence of intellectual disability in children with autism at 75%. She voiced concern regarding the quality of the data because the majority of the empirical data was published 25 to 45 years ago.

Expanded definitions of the autism spectrum have included children without intellectual disability, and ASD now includes the subgroups of autistic disorder, Asperger syndrome, and pervasive developmental disorder [8]. Recent articles emphasize diagnosis of a spectrum rather than distinct subtypes as being more appropriate [9]. For example, a study by Mayes and Calhoun questioned the validity of using cognition to distinguish between children with autism and Asperger syndrome [10].

Since the DSM-IV and inclusion of a broader definition of children with ASD, more studies have been conducted looking at the developmental profiles of children with this disorder, including their IQ, motor, and language skills. In her review, Edelson [7] found that when studies used developmental or adaptive scales, the prevalence rates of intellectual disability were higher than when measures testing IQ were used. Developmental scales assess the attainment of developmental milestones as compared to same-aged peers and are different from measures of intelligence. Rogers [11] found that low scores on developmental scales are not as predictive of later development in children with autism. 

 According to Mayes and Calhoun [12], 67% of preschool-aged children with autism had normal motor milestones but delayed speech milestones. While the preschool-aged children in their study demonstrated a gap between verbal and nonverbal IQ scores, this gap closed by the time the children were school aged. In an earlier study, 33% of children with autism who had serial IQ testing at least one year apart experienced an increase in IQ greater than 15 points [13]. Significant IQ increases have been reported for young children with ASD who receive intensive intervention [14–16].

*The Bayley Scales of Infant Development—Third Edition* [17] included a group of children with pervasive developmental disorder in the standardization process of special groups. Subjects were 70 children aged 16 to 42 months matched with a control group.

 All composite and subtest scores for the PDD group were significantly lower that those obtained by children in the matched control group. Cognition scores were one standard deviation lower for children in the PDD group, and language scores were even more significantly delayed than cognitive skills. 

In summary, the ability profiles of children with autism spectrum disorder have been highly variable in former studies. There is limited research on the comparison of the cognitive profiles of young children with and without autism who are referred for language and behavioral concerns. To further explore the cognitive profiles of children with and without autism spectrum disorder, the following research questions were asked: how do the cognitive profiles of young children with ASD differ from same-aged children seen for developmental evaluation who do not have ASD? Do young children diagnosed with ASD have higher cognitive scores than language scores on a standardized assessment tool? Are there age, gender, and socioeconomic differences between the children with ASD and those without ASD with respect to cognitive abilities?

## 2. Methods

### 2.1. Participants

This study was a retrospective chart review of children referred to the Kluge Children's Rehabilitation Center Infant and Young Child Clinic for developmental assessment during an 18-month period in 2007 and 2008. A total of 147 children ages 16 to 38 months were included in the study, with a median age of 27 months. There were 107 males (73%) and 40 females (27%). Most of the children were referred to the diagnostic clinic by either their primary physician or a family member and were seen for concerns regarding their development in areas such as language, behavior, possible autism, or global developmental delay. 

Each child was seen by an early childhood special educator and a developmental pediatrician. The majority of the children were from central Virginia and surrounding areas. The family's health insurance status—whether they had public insurance (Medicaid) or private insurance—was used as a proxy measure of socioeconomic status (SES). During the clinic visit, each family reported whether the child was receiving early intervention and/or therapy services, and the child was referred to appropriate services if they were not already enrolled in a local program. The study was approved by the Human Investigation Committee of the University of Virginia.

### 2.2. Instrumentation

Each child was given the cognitive and language tests from the *Bayley Scales of Infant Development—Third Edition* (BSID-III) by a person certified in their administration. Other tests of the BSID-III such as fine and gross motor and social and adaptive tests were not administered during the clinic visit due to time constraints. The third edition of the BSID [17] was published in 2006, and items were based on developmental research and theory that typified normal development in children from birth to 42 months. The cognitive scale contains items that assess memory, problem-solving, and counting skills. The language scale evaluates both receptive and expressive language including the child's understanding and use of words and gestures. The BSID-III was standardized using a demographically stratified sample of 1700 children. While children are compared using composite scores, a developmental age equivalent of the total raw score can also be derived. The developmental age equivalent indicates the specific age a given subtest total raw score is typically obtained by most children.

For children referred for screening of possible autism spectrum disorder, the *Childhood Autism Rating Scale* (CARS) [18] was administered by observation and parental report. The CARS is a behavior rating scale developed to assist in the diagnosis of children with autism. A 4-point rating scale ranging from 1 (normal) to 4 (severe) on 15 items yields a composite score ranking children as nonautistic, mild/moderately autistic, and severely autistic. The scale is used to observe and rate areas such as the child's relationship to people, ability to imitate, body and object use, sensory responses, adaptation to change, activity level, emotional responses, and verbal and nonverbal communication. A score of 30 meets criteria for ASD. The CARS is used frequently for diagnosis of ASD. Internal consistency of the CARS has been reported to be high, with a coefficient alpha of .94 and average interrater reliability of  .71 [19]. 

All analyses were performed utilizing SAS Version 9.1; statistical significance was defined as a *P*-value <.05. Overall comparisons between the two groups (those with ASD versus those without ASD) were made via chi-square tests for categorical variables and *t*-tests for continuous outcomes. Linear models were fit to the cognition and language outcomes, with age and group (and their interaction) as predictors. Comparisons between the two groups were then made at each of four standard ages (18, 24, 30, and 36 months) based on estimates from these models.

## 3. Results

More children referred to the diagnostic clinic were male (73%) than female. Socioeconomic status as determined by public or private insurance was not significantly different for children with ASD and without ASD, although fewer children with ASD (26%) were insured by Medicaid than children without ASD (37%). Sixty percent of the children referred to the clinic were receiving early intervention and/or therapy services at the time of the clinic visit. 

Of the 147 children referred to the clinic, 64 children were administered both the BSID-III and the CARS compared to 72 children who received only the BSID-III assessment because they were not observed to exhibit any behavioral characteristics associated with ASD. There were 54 children who were diagnosed with ASD using the CARS criteria, with a higher number of males (42) than females (12). In regards to the age of diagnosis of ASD, 42 were over 24 months of age and 12 were under 24 months of age. In addition, eleven children were uncooperative during the testing session in order to complete the BSID-III tests and were not included in the analyses. These eleven children did receive a CARS evaluation by observation and parental report. Of the 11, all but one child met criteria for ASD. 

The BSID-III cognitive and language composite scores were found to be lower overall for children diagnosed with ASD than those without ASD. The mean cognitive composite score for children with ASD was about eight points lower than those without ASD (*P* = .0006). The mean ASD language composite score was over 16 points lower (*P* < .0001). Developmental age equivalents were also lower for children with ASD in cognitive, receptive, and expressive language (Table 1). 

**Figure 1:**
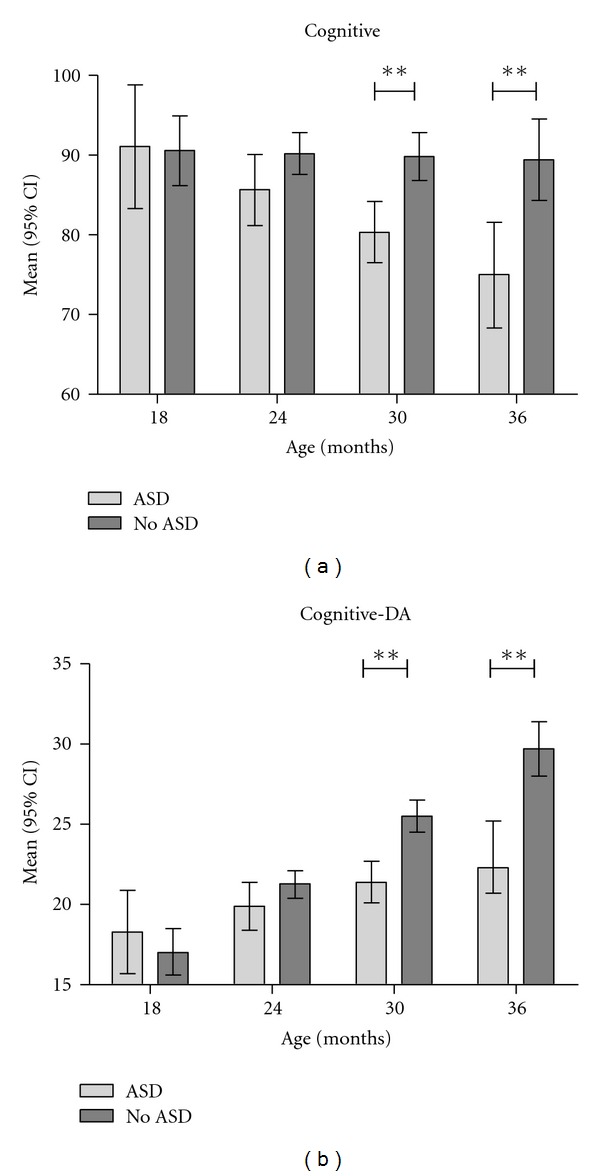
Cognition cmparisons by age, *P* < .01. DA: developmental age equivalent.

**Figure 2:**
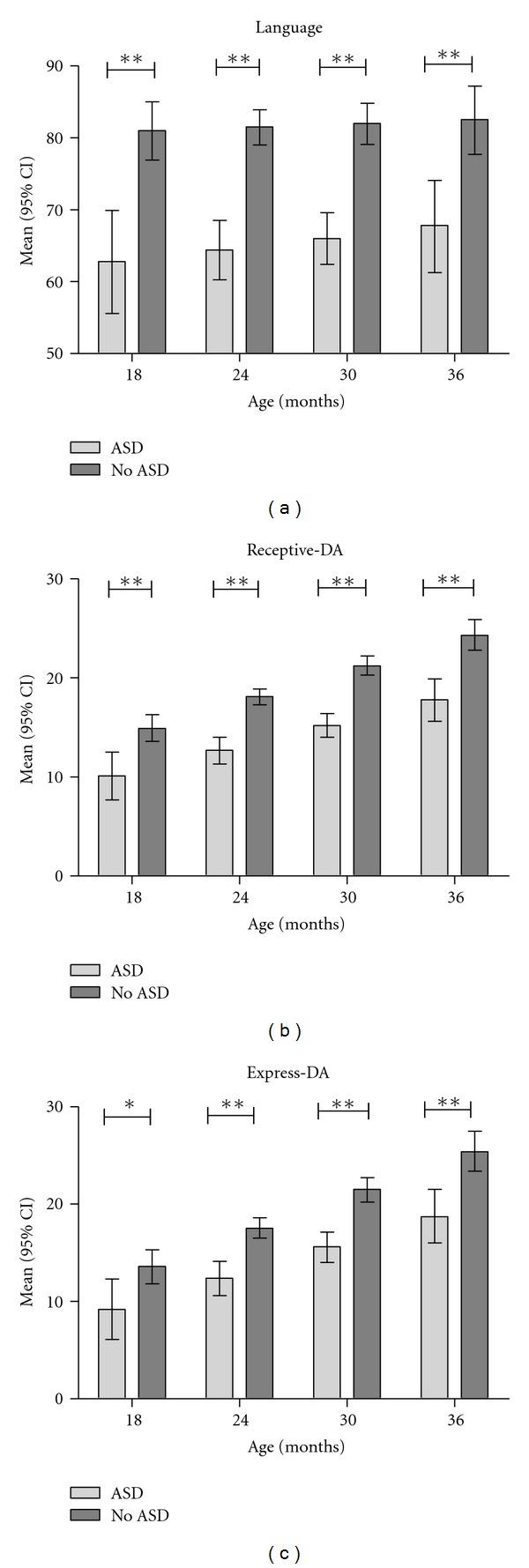
Other outcome comparisons by Age, *P < .05, **P < .01, DA: developmental age equivalent.

However, significant interactions existed between age and ASD status with respect to the cognitive outcomes (interaction *P* < .05 for both cognition outcomes). Significant cognitive differences between children with and without ASD were observed only for older children (over two years old) (Figure 1). Children with ASD who were below two years of age demonstrated low-average to average cognitive skills while older children with ASD scored in the borderline or lower range for cognitive skills. The overall differences observed for the language outcomes remained relatively consistent no matter the age (Figure 2); no significant interactions between age and group were observed for these outcomes (*P* > .4 in all cases). Including gender in the model did not impact the results.

## 4. Discussion

In the current study, the majority of young children referred to a diagnostic clinic were able to receive a standardized assessment using the BSID-III, including children who were found to have characteristics of ASD. As seen in other studies, males were more likely to be diagnosed with ASD than females. While the cognitive skills of children with ASD were generally lower than the children without ASD, many of the children with ASD scored in the average and low-average range in cognition. This relative strength in cognitive or nonverbal reasoning ability that is seen in children with ASD may provide valuable information for families or intervention agencies struggling to meet the needs of this challenging population. 

For example, the assessment of cognitive skills by a standardized tool such as the BSID-III may assist with future prognosis. Harris and Handleman found that a higher IQ (*M* = 78) and younger age (*M* = 42 months) were both predictive of placement of children with ASD in a regular classroom rather than a special education classroom following a preschool program that provided treatment using intensive applied behavioral analysis (ABA) [20]. Cognitive skills have been found to influence the age of diagnosis for ASD and the severity of autistic symptoms. Children with higher IQs are more likely to be identified at a later age [21]. Children with below normal IQs exhibit more autistic symptoms overall, including more social problems [22].

An additional finding in the current study was the discrepancy in the cognitive scores of children with ASD by age, with older children having lower cognitive scores than children below two years of age. One reason for the difference in cognitive scores between younger and older children with ASD may be that the cognitive measure of the BSID-III incorporates increasingly difficult verbal directions and responses for children two years of age and older. Language-based concepts, such as size and color discrimination and number concepts, are included in items on the cognitive test that are asked of older children. Children with ASD may have more difficulty providing the requisite verbal responses than their peers without ASD. 

As would be expected, children with ASD scored significantly lower in language skills as a group than children without ASD. Individualized assessment of language skills may assist in selecting the appropriate communication approach to use with a child. The development of a functional communication system [23] or augmentative communication systems involving signs or pictures are examples of effective strategies for children with ASD [24]. Joint attention training promotes both language and social skills development. A recent study demonstrated that joint attention and play skills could be taught and would generalize across settings and people [25]. 

 There is a growing consensus that critical components of an effective intervention program for children with ASD include early entry into a program following diagnosis, inclusion of parent training, incorporation of a high degree of structure, implementation of strategies for generalization, and low student-to-teacher ratio including one-on-one time [26]. Standardized assessment as a component of the diagnostic protocol may lead to a match of a child's abilities with the most effective intervention program. Children with excellent visual discrimination and matching skills may benefit from the systematic instruction approach utilized by TEACCH (treatment and education of autistic and related communication handicapped children) [27]. Parents with young children may feel most comfortable with the floor time approach of a relationship-based method [28].

Children who have difficulty approaching tasks may require the intensive behavior methods of the ABA approach [15]. In a review of ABA studies, Baglio found that ABA treatment resulted in consistent positive outcomes in a variety of areas including reduction in self-injurious behaviors and improvement in language, academics, daily living, and social skills [29]. Intensive behavior training has been found to be more effective than more eclectic approaches [30]. While current treatment approaches differ in philosophy, they also overlap in their incorporation of behavior management strategies and use of typical developmental milestones for curriculum development. Dempsey [31] recommended an individualized approach to educational strategies for children with autism. 

Early diagnosis and intervention services may help to ameliorate the parenting stress involved with having a child with ASD. Parents and siblings of children with ASD report experiencing more depression than those of typically developing children or even children with other disabilities [32]. Parenting a child with autism who required special service needs was found to be associated with stress [33]. A child's problems with regulation were associated with maternal stress while externalizing behaviors such as tantrums were associated with paternal stress [34]. 

Early identification of children with ASD will need to be addressed by a variety of health care providers, including primary care physicians, pediatricians, and public health nurses. Referral for differential diagnosis should be completed as soon as concerns are raised. Because of the ongoing needs of families with children with ASD, health care providers will need to have knowledge regarding community resources that may be able to assist the family including early intervention programs, early childhood special education programs through the local public schools, child and family counseling services, and parent support groups.

## 5. Limitations of the Study

The current study is limited by the retrospective nature of the data collection. The subjects were children who were referred to a diagnostic developmental clinic and therefore may not represent a broader sample of the population. The assessment of the children was limited to cognitive and language domains of the BSID-III so that other areas of development were not included that may have been useful for instructional or diagnostic purposes. The children were seen only once by the educator so that longitudinal information was not available. The children were young and may have significantly different developmental profiles in the future due to maturation or intervention. It should be noted that the educator conducting the evaluations is also one of the authors of this study.

## 6. Conclusions

The current study found that young children being screened for possible ASD were able to be evaluated using a standardized measure such as the BSID-III. As observed in other studies, males were much more likely to be diagnosed with ASD than females. Children who were over two years of age were more likely to be diagnosed with ASD and demonstrated lower cognitive scores than younger children or children without ASD. While the cognitive skills of children with ASD were slightly lower than children without ASD, specific abilities were observed that could be used to match a child's skills with treatment options. Language skills were significantly more impaired and predictive of a diagnosis of ASD, but children without ASD also exhibited a high rate of language delay and would benefit from early intervention services. The developmental profiles from standardized assessments may be used for referral to appropriate educational programs and provide valuable information for intervention strategies. While all children with language delays will benefit from referral to services, children with ASD will require more intensive services tailored to their specific strengths and challenges.

## Figures and Tables

**Table 1:** Overall Comparisons.

Variable	Overall	Children with ASD	Children without ASD	*P*-value*
*N*	147	54	93	
Categorical variables (%)				
Male	72.8%	77.8%	69.9%	.3004
Insured by Medicaid	33.6%	26.5%	37.4%	.1995
Continuous variables (Mean ± SD)				
Age (months)	26.5 ± 5.4	27.6 ± 5.1	25.9 ± 5.6	.0730
Cognitive composite score	87.5 ± 12.7	82.2 ± 12.4	90.1 ± 12.1	.0006
Language composite score	76.3 ± 13.4	65.4 ± 10.7	81.6 ± 11.2	<.0001
Cognitive-DA	22.1 ± 5.2	20.9 ± 4.3	22.7 ± 5.5	.0579
Receptive-DA	17.5 ± 5.1	14.2 ± 4.6	19.1 ± 4.6	<.0001
Express-DA	17.4 ± 5.2	14.3 ± 5.6	18.8 ± 5.9	<.0001

*Group comparisons were performed via chi-squared tests (categorical variables) and *t*-tests (continuous outcomes).

DA: developmental age equivalent.
